# Sarcopenic Obesity in Older Adults: Findings From the National Health and Aging Trends Study

**DOI:** 10.1093/geroni/igab046.2351

**Published:** 2021-12-17

**Authors:** Kathleen Dondero, Jason Falvey, Brock Beamer, Odessa Addison

**Affiliations:** 1 University of Maryland - Baltimore, Baltimore, Maryland, United States; 2 Baltimore Veterans Affairs Medical Center, BALTIMORE, Maryland, United States; 3 University of Maryland School of Medicine, Baltimore, Maryland, United States

## Abstract

Sarcopenic obesity increases risk for dysmobility and loss of independence, (Gandham et al., 2021). However, the national burden of sarcopenic obesity and the resultant impacts for older adults has yet to be described. Within a nationally representative sample from the National Health and Aging Trends Study (NHATS), 2066 community-dwelling older adults were obese, representing 12,136,374 individuals in the United States, or 31.8% of all community dwelling older adults. Based on the European Working Group definition, 18% of the obese older adults were sarcopenic. Sarcopenic obese older adults were more likely to have fallen in the last month and been hospitalized over the prior year. After adjusting for age and sex, sarcopenic obese older adults were 3.7 times more likely (95% CI 2.2-5.0) to have 2 or more comorbid conditions and frailty was 6.4 times more likely (95% CI 4.4-9.5) compared to nonsarcopenic obese older adults. Sarcopenic obese older adults were also more likely to have 1+ ADL disabilities (OR 3.7; 95% CI 2.5-5.4). Further, they were more likely to be socially isolated (OR 2.1; 95% CI 1.3-3.2) and report food insecurity (OR 1.5; 95% CI 0.8-2.9). These findings suggest older adults with obesity and sarcopenia have higher rates of geriatric vulnerabilities, which might indicate a need for caution when recommending weight loss alone as an intervention. A more comprehensive intervention may be necessary to address social and physiological risks. Future studies should examine whether early intervention in sarcopenic obese older adults can reduce chronic health risk and preserve independence.

